# Towards A Molecular Understanding of The Cannabinoid Related Orphan Receptor GPR18: A Focus on Its Constitutive Activity

**DOI:** 10.3390/ijms20092300

**Published:** 2019-05-09

**Authors:** Noori Sotudeh, Paula Morales, Dow P. Hurst, Diane L. Lynch, Patricia H. Reggio

**Affiliations:** 1 Department of Chemistry and Biochemistry, University of North Carolina at Greensboro, Greensboro, NC 27412, USA; n_sotoud@uncg.edu (N.S.); dphurst@uncg.edu (D.P.H.); lynchdl@gmail.com (D.L.L.); 2 Instituto de Química Médica, Consejo Superior de Investigaciones Científicas, 28006 Madrid, Spain; paula.morales@iqm.csic.es

**Keywords:** GPR18, cannabinoids, homology model, G protein-coupled receptor, constitutive activity, molecular dynamics

## Abstract

The orphan G-protein coupled receptor (GPCR), GPR18, has been recently proposed as a potential member of the cannabinoid family as it recognizes several endogenous, phytogenic, and synthetic cannabinoids. Potential therapeutic applications for GPR18 include intraocular pressure, metabolic disorders, and cancer. GPR18 has been reported to have high constitutive activity, i.e., activation/signaling occurs in the absence of an agonist. This activity can be reduced significantly by the A3.39N mutation. At the intracellular (IC) ends of (transmembrane helices) TMH3 and TMH6 in GPCRs, typically, a pair of oppositely charged amino acids form a salt bridge called the “ionic lock”. Breaking of this salt bridge creates an IC opening for coupling with G protein. The GPR18 “ionic lock” residues (R3.50/S6.33) can form only a hydrogen bond. In this paper, we test the hypothesis that the high constitutive activity of GPR18 is due to the weakness of its “ionic lock” and that the A3.39N mutation strengthens this lock. To this end, we report molecular dynamics simulations of wild-type (WT) GPR18 and the A3.39N mutant in fully hydrated (POPC) phophatidylcholine lipid bilayers. Results suggest that in the A3.39N mutant, TMH6 rotates and brings R3.50 and S6.33 closer together, thus strengthening the GPR18 “ionic lock”.

## 1. Introduction

GPR18 is an orphan cannabinoid-like receptor that belongs to the class A family of G-protein coupled receptors (GPCRs). GPR18 is highly expressed in the central nervous system and lymphoid tissues and it displays high constitutive activity in cells. Its modulation has been associated with numerous physiopathological processes, such as cellular migration, immunomodulation, sperm physiology, cardiac physiology, obesity, intraocular pressure, pain, and cancer, among others [1,2,3,4,5].

The lipid derivative, N-arachidonoyl glycine (NAGly), has been proposed as the putative endogenous ligand for this receptor [6,7,8]. Likewise, the endogenous polyunsaturated fatty acid metabolite, Resolvin D2 (RvD2), implicated in inflammatory processes, has also been reported to target GPR18 [9]. Nonetheless, the International Union of Pharmacology (IUPHAR) still categorizes GPR18 as an orphan GPCR due to the lack of consistency amongst reports [10,11] and the existence of limited in vivo evidence [12,13,14]. 

GPR18 shows relatively low sequence homology with the cannabinoid receptors type 1 and type 2 (CB1 and CB2) (~13% and 8%), yet a sub-set of endogenous, phytogenic, and synthetic cannabinoid ligands have been shown to modulate GPR18 [14]. This fact has prompted numerous studies to understand the relationship between GPR18 and the endocannabinoid system, which comprises the CB1 and CB2 receptors; endogenous ligands, anandamide, and 2-arachidonoylglycerol (2-AG); their associated enzymes; and transporter. 

GPR18 signaling pathways are still not fully explored due to the lack of pharmacological tools, challenging heterologous expression and high intracellular receptor expression [3,15]. Coupling to Gαi/o and Gαq has been reported for GPR18 [5,6,7,8,16], whereas GPR18 did not couple to other G proteins tested, including Gαs, Gαz, and Gα15 [10]. Several research groups have found that GPR18 has high constitutive activity [3,15]. For instance, Glass and coworkers observed that introduction of an A3.39N mutation triggered a reduction in constitutive activity, as well as a higher steady-state surface receptor expression [15]. The role of the residue located at position 3.39 has been suggested to be relevant at the sodium binding pocket and to be critical to the control of constitutive activation at specific GPCRs, such as the chemokine receptors [17,18,19].

At the intracellular (IC) end of class A GPCRs, an “ionic lock” formed typically by R3.50 and D/E6.30 forms a salt bridge that holds the receptor in its inactive state. Breaking of this lock creates an opening to allow coupling with G-protein, i.e., signaling. While GPR18 has an arginine at 3.50, it lacks a negatively charged residue at the end of TMH6. Instead, GPR18 has a hydrogen bonding residue, S6.33, at this position that interacts with R3.50. In this paper, we test the hypothesis that the high constitutive activity of GPR18 is due to the weakness of the GPR18 “ionic lock” and that the A3.39N mutation strengthens this lock and thereby reduces constitutive activity. To this end, we have constructed homology models of wild-type (WT) GPR18 and the A3.39N mutant in their inactive states. These models were used to conduct molecular dynamics simulations of WT GPR18 and the A3.39N mutant in fully hydrated 1-palmitoyl-2-oleoyl-sn-glycero-3-phosphocholine (POPC) lipid bilayers. Our results suggest that the presence of N3.39 can lead (via changes in the sodium binding pocket) to a TMH6 rotation that brings R3.50 and S6.33 closer together, thus strengthening the “ionic lock” in the A3.39N mutant and reducing constitutive activity.

These molecular modeling studies provide structural insights into GPR18 and its activation. Knowledge gained in the present study should provide a structural basis for mutation studies of GPR18 and the development of novel ligands for GPR18. 

## 2. Results and Discussion

### 2.1. GPR18 Model Development 

**Figure 1:**
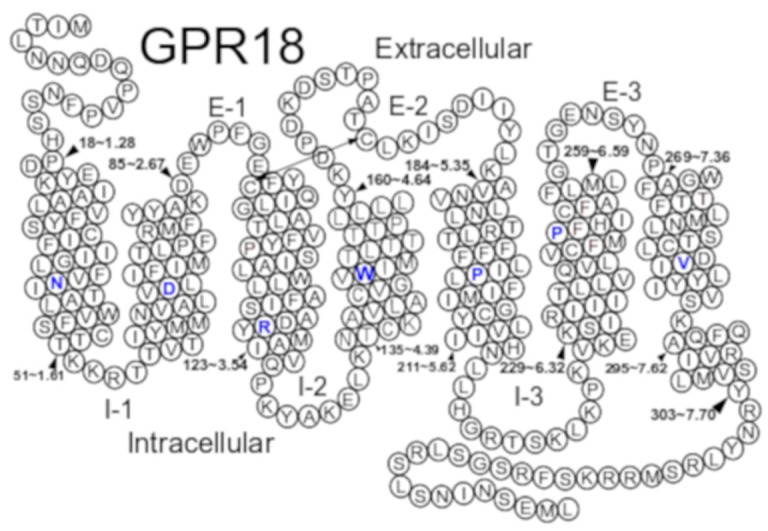
Helix net of the human GPR18 sequence. Among class A GPCRs, the most highly conserved residue in each helix is colored blue. GPR18 has a proline at P3.36, it lacks the highly conserved proline at position 7.50, bearing a valine at that position instead. A key disulfide bridge between the extracellular loop 2 (EC-2) and the top of TMH3 is indicated by a double headed arrow. Black arrowheads indicate specific residue numbers in the GPR18 sequence (absolute and Ballesteros-Weinstein numbering).

Our GPR18 homology model of GPR18 was constructed using the X-ray crystal structure of the delta opioid receptor (DOR) at a 1.8 Å resolution as a template (PDB identifier: 4N6H). The rationale for the choice of template is provided in the Methods section. Upon mutation of the crystal template, the initial model was refined on the basis of sequence divergences between GPR18 (Figure 1) and the DOR crystal structure. Conformational differences in specific transmembrane helices were investigated using the simulated annealing/Monte Carlo (MC) conformational analysis method of conformational memories (CM) as described in the Methods.

#### 2.1.1. TMH3 

In the GPR18 sequence, there is a P3.36 in TMH3 (transmembrane helix 3) that is unique. Since the presence of a proline introduces a kink in the helix (well documented in previous reports) [20], the CM method was used to explore the possible conformational space of TMH3. To achieve this goal, an initial regular α-helix was built and then variations around the proline kink region were introduced. This region includes the residues from P3.36 to T3.32. The range of variations was: −120° < φ < 40°, −70° < ψ < 0° and −160° < ω < 160°. These values were extracted from the Protein Data Bank [21]. Finally, the 103 structures generated by CM were superimposed from P3.36 to Q3.56. Results were compared with the template. Although TMH3 in the DOR crystal structure lacks a P3.36 (M3.36 instead), it is bent in this region with an average bend angle of 16.5°, calculated using the ProKink program [22]. This bend is structurally explained by the presence of T3.38 in a gauche minus (g^−^ = 0° < χ1 < 120°) dihedral. Average Ser/Thr kinks measured in alpha helices are usually around 12° [23]. CM outputs suggested that GPR18 TMH3 with its P3.36 actually has a proline kink angle of 16.8°, similar in magnitude to the Ser/Thr induced bend in TMH7 of DOR. 

#### 2.1.2. TMH7 

GPR18 lacks the TMH7 NPXXY motif. Instead, it has DVXXY. Prolines are hinge residues capable of bending helices at specific positions [22]. It may seem that the lack of P7.50 will lead to a straight helix. However, our conformational studies led us to the observations detailed below. On the one hand, incorporating a straight TMH7 in the TMH bundle created an empty space on the extracellular (EC) side of the bundle that cannot be filled by EC loops (specifically EC2 and 3), allowing more water to enter the orthosteric binding pocket than would be expected for a GPCR that binds lipid-like ligands. Second, a straight TMH7 changes the position of some key residues. For example, N7.43 is typically a binding pocket residue, but if TMH7 is straight, its Cα position will face the lipid. Residue 7.39 (T7.39) has been found in several GPCRs to be a ligand interaction site and therefore, normally, 7.39 is more accessible to the binding crevice. However, in a straight TMH7 helix, T7.39 faces towards TMH1 and away from the typical ligand binding pocket. Finally, in a straight TMH7, Y7.53 deviates from its normal position in the sodium binding pocket [19] (the region with polar residues, including N1.50, D2.50, S3.39, N7.49, and Y7.53, that are coordinating directly with sodium ion or water molecules). 

While TMH7 lacks a proline at 7.50, there are other residues in TMH7 that may induce a helix distortion/hinge similar to a proline. Ballesteros et al. [23] showed that serines and threonines in alpha helices can act as hinge residues. The bending produced by each of these residues is produced by an intrahelical hydrogen bond between the backbone carbonyl oxygen (at position i-4 for an alpha helix and i-3 for a 3/10 helix) and the O atom of the Ser or Thr in a χ1 g- dihedral. In GPR18 TMH7, residues S7.45 and T7.46 deserve particular attention because of their close location to the common P7.50 in other GPCRs. Therefore, we used the CM method to explore the effect of S7.45 and/or T7.46 on TMH7 conformations. This study explored g^−^ /g^+^, g^+^/g^−^, and g^−^/g^−^ combinations of S7.45 and T7.46, respectively, by variation of the χ1 dihedral of these two residues to the chosen value. The variation range was the same as that used for the proline kink in TMH3 for the φ, ψ, and ω dihedrals. Altogether, the g^−^/g^−^ combination mimicked best the kink produced by P7.50 in DOR. Among the low-free energy TMH7 conformers obtained using CM, the TMH7 helix that fit in the bundle without van der Waals overlaps with residues on other TMHs was selected for the GPR18 model. This TMH7 has a helix bend angle of 14.4°, a wobble angle of −16.2°, and a face shift of 26.4° at S7.45 and has a helix bend angle of 15.7°, a wobble angle of −106.5°, and a face shift of 32.6° at T7.46. 

#### 2.1.3. GPR18 Activation 

The binding of an agonist and subsequent receptor activation is accompanied by significant conformational changes in the receptor inactive state structure. Constitutively activated GPCRs do not need the presence of an agonist to achieve the active state (R*). The main structural changes that define the R* bundle occur in the cytoplasmic end of the receptor. One of the crucial features is the outward movement of TMH6 from the receptor core. This movement causes the breakage of the “ionic lock” (R3.50 and S6.33 interaction, further detailed below), increasing the distance between the IC ends of TMH6 and TMH3. This movement generates an intracellular opening that allows G-protein interaction. P6.50 determines the flexible hinge region that mediates the transition from R to R* [24]. To create our activated bundle from the GPR18 inactive state R model, a CM analysis was performed in the hinge region of P6.50 to V6.46. From the obtained outputs, a less kinked TMH6 conformer (bend angle: 22°) was selected for TMH6 in the R* state model. This TMH6 conformer pulls away from the TMH bundle on the IC side, ensuring that the TMH3-TMH6 “ionic lock” is broken. The resultant bundle was then minimized as described in the Methods section.

#### 2.1.4. Ionic Lock and Toggle Switch 

TMH6 plays an important role in the transition between the inactive and activated states. When the receptor is activated, the intracellular end of TMH6 moves away from transmembrane helix bundle, opening an intracellular space for coupling with the G-protein. This mechanism is mediated by two important switches that are common between TMH3 and 6. The first one is the “ionic lock” at the IC ends of TMH3 and TMH6. In most class A GPCRs, the “ionic lock” is a salt bridge between R3.50 and D/E6.30. In GPR18, the ionic lock is actually a stable hydrogen bond formed between R3.50 and S6.33 (O-N distance = 2.9 Å; NH-O angle of 164°). The role of the “ionic lock” is to limit the relative mobility of the cytoplasmic ends of TMH3/6 in the inactive state, and by disruption in the active state, leads to the formation of an opening at the bottom of the receptor into which the G-protein inserts its α-5 helix to couple with the receptor. 

**Figure 2:**
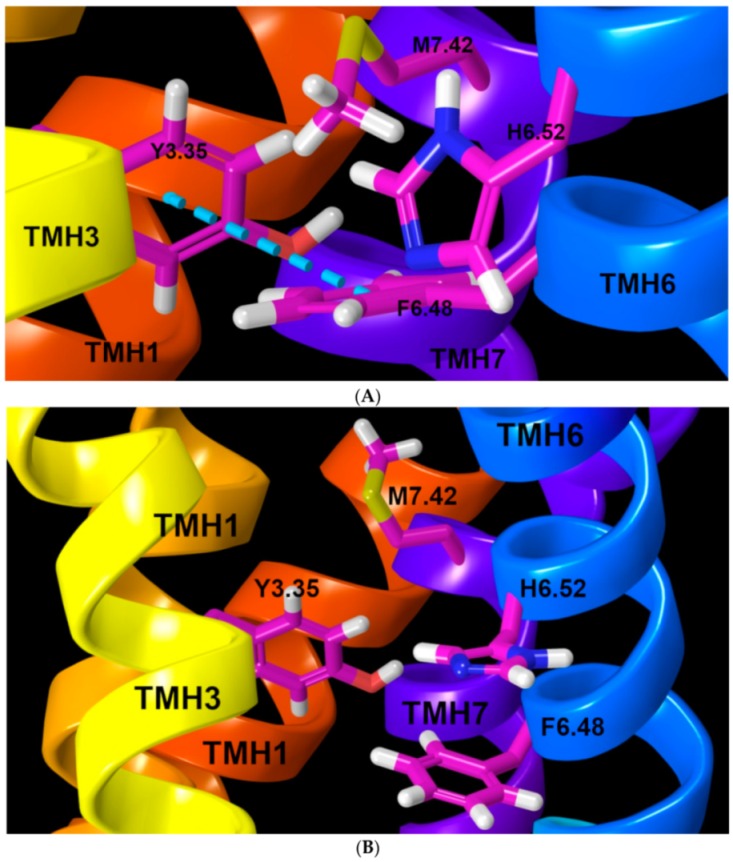
(**A**) The toggle switch in GPR18 R state: M7.42/F6.48 and Y3.35/F6.48 interactions (blue dashed line) keep the F6.48 χ1 angle in a g+ rotamer, which retains the H6.52 χ1 in g+ in the inactive state (R) model. Key residues are highlighted in magenta thin tubes. (**B**) The toggle switch in the activated state: the M7.42/F6.48 and Y3.35/F6.48 interactions have broken, causing the F6.48 χ1 to switch to a trans rotamer. This synergistically leads H6.52 to adopt a χ1 trans conformation in the R* model. Key residues are highlighted in magenta thin tubes.

A second key structural feature is the “toggle switch”. The toggle switch is a pair (trio or quartet) of residues, one of which is W/F6.48 (of the CWXP flexible hinge motif) and the other(s), is (are) typically an aromatic or bulky residue that forms an interaction or stack with W/F6.48, stabilizing it in a χ1 = g+ conformation. In GPR18, the toggle switch is formed by F6.48, M7.42, and H6.52 and Y3.55 (see Figure 2A). When an agonist enters the GPR18 binding pocket, it triggers a F6.48 χ1 change from g+ to trans. This conformational change straightens TMH6 and breaks the ionic lock. Since antagonists/inverse agonists stabilize the inactive state of the receptor, an antagonist/inverse agonist should block the F6.48 χ1 transition from g+ to trans, thus keeping GPR18 in its inactive state. The highly conserved F6.44XXCWXPXF6.52 motif is replaced by V6.44XXCFXPXH6.52 in GPR18. Moreover, the lack of a bulky residue, like M3.36 (in GPR55), or a F3.36 (like CB1 and CB2) [25] directly across from F6.48 makes it difficult to control the toggle switch in GPR18. Since there is a P3.36 at this position, there is a lot of room for F6.48 to change its χ1 conformation from g+ to trans.

Our GPR18 model suggests that Y3.35 and M7.42 directly, and H6.52 indirectly, participate in the GPR18 toggle switch. The current model shows that M7.42 packs the terminal CD methyl carbon against the aromatic ring face of F6.48 by interacting through a favorable Met/aromatic interaction to hold F6.48 in its χ1 = g+ dihedral (M7.42 CG, SD, and CE atom distances to the centroid of F6.48 are 4.26 Å, 5.01 Å, and 3.93 Å, respectively). In addition, Y3.35 presents an aromatic face to the aromatic edge of F6.48 in a tight tilted-T aromatic stack (centroid to centroid distance = 5.2 Å; angle of 58°). H6.52 is i + 4 to F6.48 and is sterically required to switch trans synergistically when F6.48 undergoes the χ1 g+ to trans rotameric change (Figure 2B). These residues are also in a tilted-T aromatic stack (centroid to centroid distance = 5.99 Å; angle of 75.9°). Taken together, our model predicts that an antagonist should promote M7.42 to pack against F6.48. An antagonist could also hold H6.52 in a χ1 = g+ to promote antagonism. However, an agonist could insert between Y3.35 and F6.48, while moving M7.42 away from F6.48. 

### 2.2. Molecular Dynamics: Structural Insights into Its Constitutive Activation 

GPR18 is a highly constitutively active receptor that produces low cell surface expression [15]. A GPR18 A3.39(108)N mutant has been reported to reduce this constitutively cycling phenotype, resulting in higher steady-state surface receptor expression [15]. As a test of our newly developed GPR18 models, we undertook a study of the effects of an A3.39(108)N mutation on the ability of the mutant to activate. To do this, we performed molecular dynamics simulations of (1) the wild-type (WT) GPR18 inactive state and (2) the mutant A3.93(102)N inactive state. Each inactive state model was embedded in a fully hydrated 1-palmitoyl-2-oleoyl-sn-glycero-3-phosphocholine (POPC) lipid bilayer and each was simulated for 700 ns, with the first 100 ns required for equilibration. Full details of MD simulations are provided in the Molecular Dynamics (MD) section. In these simulations, helix8 was placed parallel to the plane of the membrane at the lipid/water interface. In addition, a sodium ion was positioned in the putative sodium ion binding site to stabilize the inactive state of the receptor. The role of sodium ions inside the GPCRs has been well documented [19,24,26,27]. 

#### 2.2.1. Ability to Maintain “Ionic Lock” and Arginine Cage 

**Figure 3:**
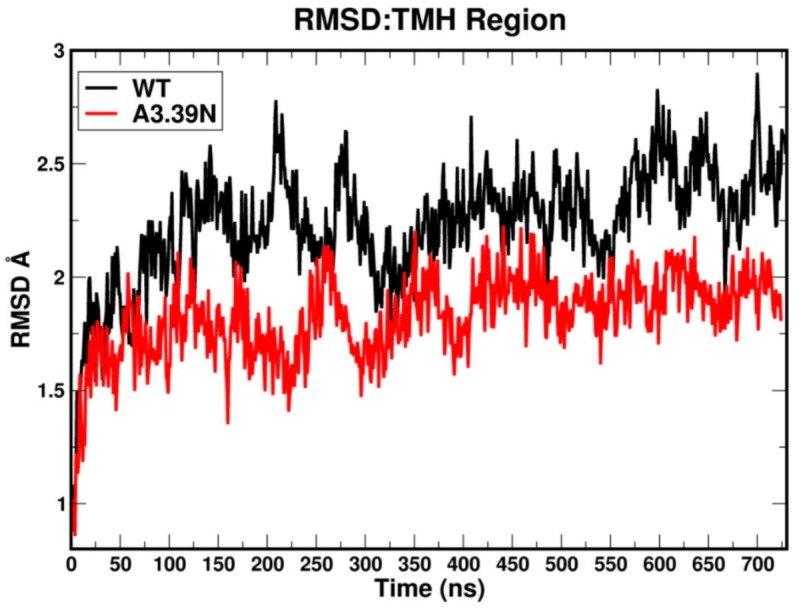
This figure shows the RMSD vs. simulation time plots for the WT GPR18 R state (black) and the A3.93N mutant R state (red). The RMSD levels out by 100 ns in both simulations. So, the first 100 ns correspond to the equilibration phase and the next 600 ns are considered production.

**Figure 4:**
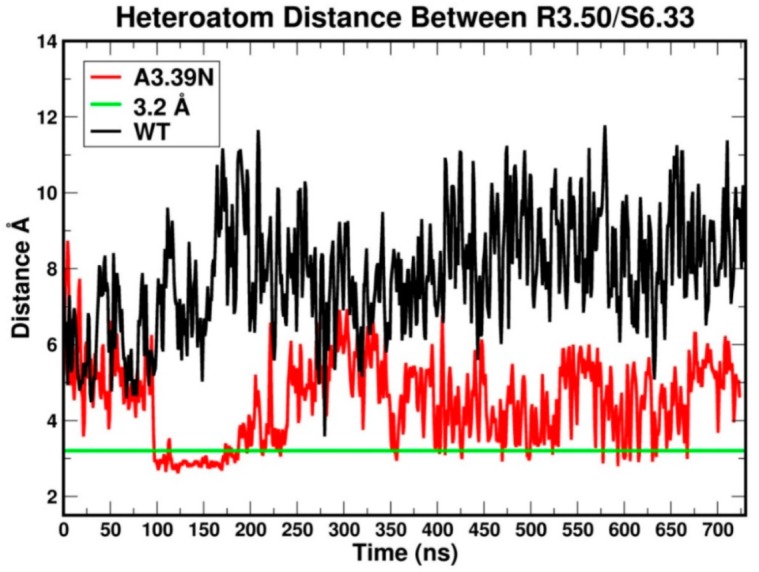
This plot evaluates the stability of the “ionic lock” over the course of the WT (black) and A3.93N (red) trajectories. The green line shows the maximum distance between heteroatoms for a hydrogen bond to exist (3.2 Å). The first 100 ns correspond to the equilibration phase. The next 600 ns are considered the production phase.

The RMSD (root mean square deviation) plots for the WT (black) and A3.39N mutant [28] are shown in Figure 3. The WT system shows gradual fluctuations and quick stabilization with a final RMSD less than an average of 2.5 Å, while the A3.39N mutant also shows gradual fluctuations and quick stabilization with a final RMSD less than an average of 2.0 Å. Figure 4 illustrates the heteroatom to heteroatom distance for the R3.50-S6.33 “ionic lock” pair over the course of the WT (black) and A3.93N [28] trajectories. The green line in Figure 4 marks the maximum heteroatom to heteroatom distance for a hydrogen bond to exist (3.2 Å). Points along the trajectory where the plot falls under the green lines are regions where the R3.50/S6.33 hydrogen bond is formed. As the molecular dynamics (MD) results in Figure 4 show, the “ionic lock” in WT breaks immediately and never re-forms. In contrast, the “ionic lock” hydrogen bond for the A3.39N mutant first forms around 100 ns and persists to 225 ns, it then “flickers” on and off during the rest of the trajectory until very near the end at ~670 ns. The fact that the R3.50-S6.33 hydrogen bond in the WT trajectory never re-forms once broken, but the same hydrogen bond in the A3.39N mutant forms, breaks, and re-forms over the course of the trajectory is a reflection of the strength of the hydrogen bond between a charged and uncharged partner, which is not as strong as the R3.50-D/E6.30 salt bridge “ionic lock” found in many GPCRs. However, there clearly must be a difference between the IC domains in the WT vs. mutant, since the mutant manages to form the “ionic lock” at least some of the time. Taken together, the MD simulations suggest that the WT receptor is extremely constitutively active, while the mutant is less so. They also suggest that the A3.39N mutant should show a decrease in basal activity. These two results are consistent with the GPR18 literature [15]. 

**Figure 5:**
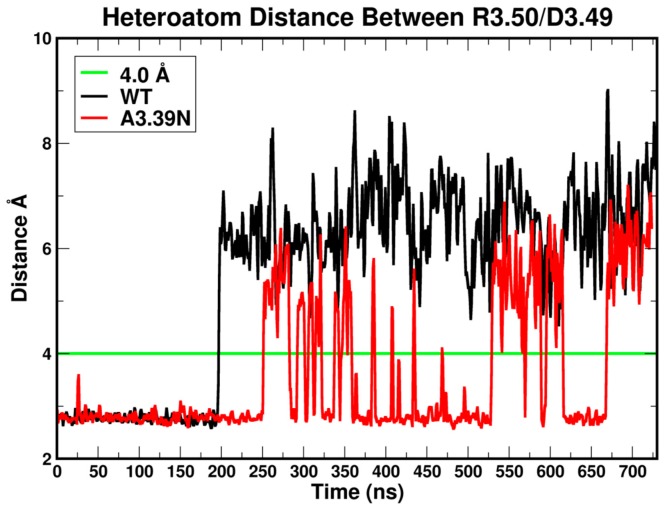
Heteroatom distance between R3.50 and D3.49 in the WT GPR18 (black) and the A3.39N mutant (red). The green line at 4 Å marks the maximum salt bridge distance between heteroatoms. The first 100 ns correspond to the equilibration phase. The next 600 ns are considered the production phase.

It has been proposed that in the inactive state of class A GPCRs, R3.50 is located in an “arginine cage,” in which it forms an intra-helical salt bridge interaction with D3.49 [29]. Figure 5 illustrates plots of the heteroatom to heteroatom distance between R3.50 and D3.49 for WT GPR18 (black) and the A3.39N mutant (red) [28]. The green line at 4.0 Å marks the maximum heteroatom-to heteroatom salt bridge distance. This plot shows that an R3.50-D3.49 salt bridge exists for a large amount of time for the mutant, but this salt bridge exists for WT until 200 ns and is thereafter broken. 

**Figure 6:**
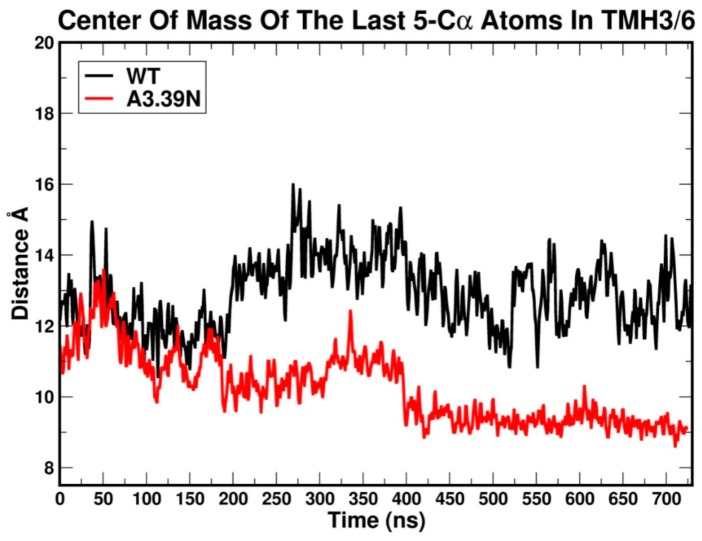
Average distance between Cα atoms of the last five cytoplasmic residues in TMH3 (Y3.51 to V(124) and TMH6 (K6.30 to I6.34) in wild type GPR18 WT (black) and the A3.39N mutant (red). The first 100 ns correspond to the equilibration phase. The next 600 ns are considered the production phase.

These results suggest that the A3.39N mutant receptor has a more stabilized arginine cage than WT. Since R3.50 has to be in a specific position in the arginine cage to interact with D3.49, this position may promote R3.50′s ability to also hydrogen bond to S6.33. Figure 6 shows a plot of the average distance between Cα atoms of the last five residues on the cytoplasmic side of TMH3 and of TMH6 for WT (black) and the A3.39N mutant [28]. As depicted in Figure 6, the distance of the centers of mass of the last five cytoplasmic end residues of TMH3 (residues Y3.51 to V(124)) and TMH6 (residues K6.30 to I6.34) clearly reveals that in the A3.39N mutant receptor, the cytoplasmic ends of TMH3 and TMH6 are closer during the course of the trajectory than those of the WT receptor. In fact, after 200 ns, the bottom of the WT receptor appears to be opened, suggesting that it has activated. The tendency of the WT receptor to have its ionic lock broken and the TMH3 and TMH6 IC ends to move away from one another is consistent with the very high constitutive activity reported for GPR18 [15]. 

#### 2.2.2. The Sodium Binding Pocket 

**Figure 7:**
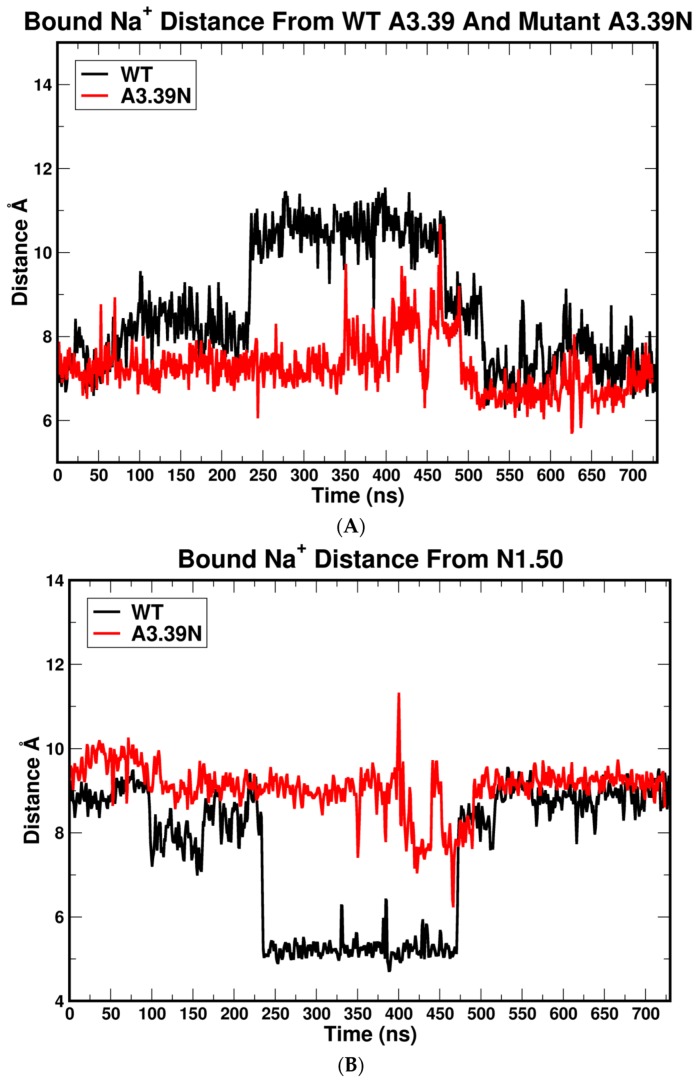
(**A**) The distance between the sodium ion and N3.39 in the A3.39N mutant (red) and A3.39 in WT (black). The first 100 ns correspond to the equilibration phase. The next 600 ns are considered the production phase. (**B**) The distance between the sodium ion and N1.50 in the A3.39N mutant (red) and WT GPR18 (black). The first 100 ns correspond to the equilibration phase. The next 600 ns are considered the production phase.

**Figure 8:**
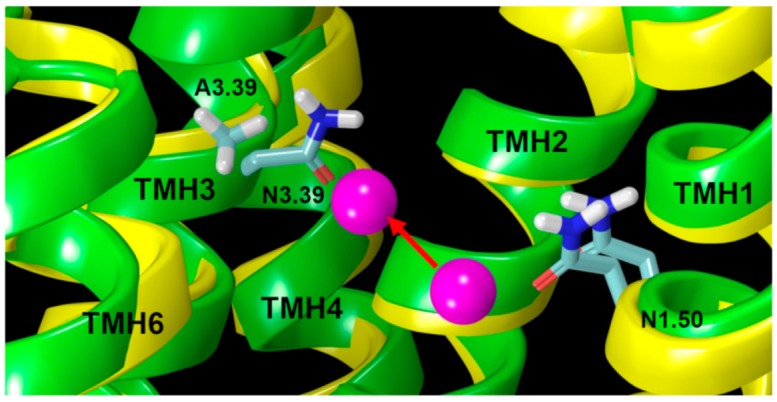
Comparison of the sodium ion (magenta sphere) position in the WT bundle (yellow) and A3.39N bundle (green). The lower sodium ion is its position in the WT receptor (yellow bundle); while the upper sodium ion position is that in the A3.39N mutant (green bundle). The red arrow shows the shift of the sodium ion position towards the EC side of the receptor. Key residues are highlighted in light blue.

To explain the relationship between the A3.39N mutation and GPR18 constitutive activity, the position of A3.39 in the two simulations previously described was analyzed. Results from prior studies have shown that in many GPCRs, sodium ions stabilize the inactive state of the receptor by interacting with two highly conserved residues, D2.50 and S3.39, and three water molecules [19]. Interestingly, the GPR18 sequence is different from most class A GPCRs in this region as it lacks the highly conserved polar residue, S3.39 (WT GPR18 has an A3.39 instead). The second important difference in the GPR18 sequence is that unlike 86% of class A GPCRs, GPR18 lacks the NPXXY motif in TMH7 (N7.49-Y7.53). Instead, it has DVXXY (D7.49-Y7.53). The presence of a negatively charged residue, D7.49, instead of a neutral one, N7.49, and the lack of a polar residue, like S3.39, might cause a shift of the sodium ion position toward the cytoplasmic end of the receptor by coordination with two aspartic acid residues, D2.50 and D7.49 (instead of D2.50 and S3.39). These drastic sequence differences in the sodium binding pocket region indicate that it is more likely that the sodium ion is located in a lower place than normally observed in other receptors. In fact, our MD results support this hypothesis. The distance between the sodium ion and the Cα atom of N3.39 in the A3.39N mutant is shorter than the distance between the sodium ion and the Cα atom of A3.39 in WT (Figure 7A and Figure 8). 

On the other hand, the distance between the Cα atom of N1.50 and the sodium ion increased in the A3.39N mutant (Figure 7B and Figure 8). Therefore, the sodium ion has moved towards the EC side in the A3.39N mutant. This is due to the hydrophilicity of residue N3.39, which contributes more in binding to the sodium ion than the WT hydrophobic residue (A3.39) would contribute. The sodium ion is pulled approximately 3.0 Å up towards the EC region of the receptor (Figure 8). 

**Figure 9:**
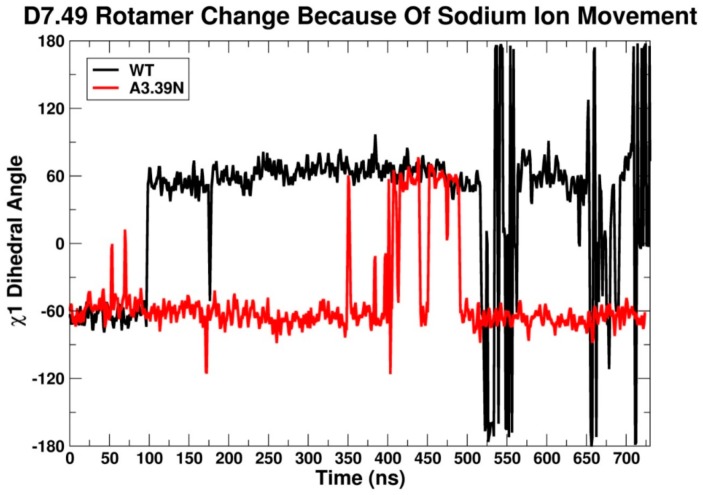
χ1 rotamer conformational change in D7.49 in WT GPR18 (black) and the A3.39N mutant (red) due to sodium ion movement. The first 100 ns correspond to the equilibration phase. The next 600 ns are considered the production phase.

**Figure 10:**
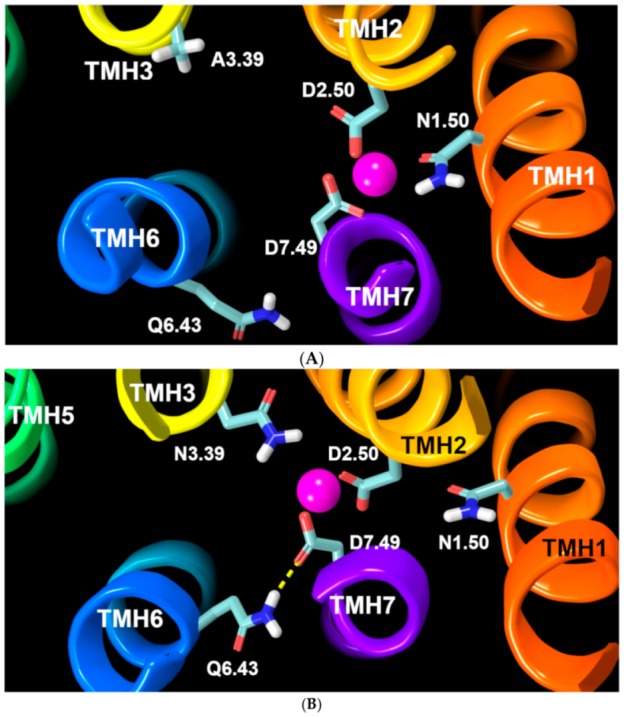
(**A**) WT GPR18 bundle: The sodium ion (magenta sphere) is coordinated between D7.49, D2.50, and N1.50 (highlighted in light blue tubes). To achieve this, D7.49 needs to break its hydrogen bond with Q6.43. (**B**) A3.39N mutant bundle: The sodium ion (magenta sphere) is coordinated between D7.49, D2.50, N1.50, and N3.39 (highlighted in light blue tubes) so that it is possible for D7.49 to keep the hydrogen bond with Q6.43 (yellow dashed line).

The other important aspect of the unique sodium ion binding pocket in GPR18 is the conformation of D7.49. MD results after 100 ns show that in the WT receptor, the D7.49 χ1 torsion tends to adopt a g^−^ rotamer conformation (+55°, average), instead of a normal g+ rotamer conformation (Figure 9). This is because in the WT system, two negatively charged residues, D2.50 and D7.49, are strong enough to pull the sodium ion down below the D7.49 carboxylic group side chain. Thus, the ideal conformation in the WT receptor for D7.49 to easily access the sodium ion and directly interact with it is a χ1 = g^−^ conformation (Figure 10A, Movie S1). Conversely, the A3.39N mutant simulations show that D7.49 retains its g+ conformation, (−55° on average) (Figure 9). Since in the A3.39N mutant, the sodium moves up (because of the presence of N3.39, Figure 8), the carboxylic acid group of D7.49 follows the ion upward so that they can interact, with the D7.49 assuming a g+ χ1 value (Figure 10B, Movie S3). The detailed analysis shows that D7.49 is able to form an additional important inter-helical hydrogen bond with Q6.43 in the A3.39N mutant (Figure 10B, Movie S3). In contrast, this inter-helical hydrogen bond is broken in the WT bundle (Figure 10A, Movie S1). 

**Figure 11:**
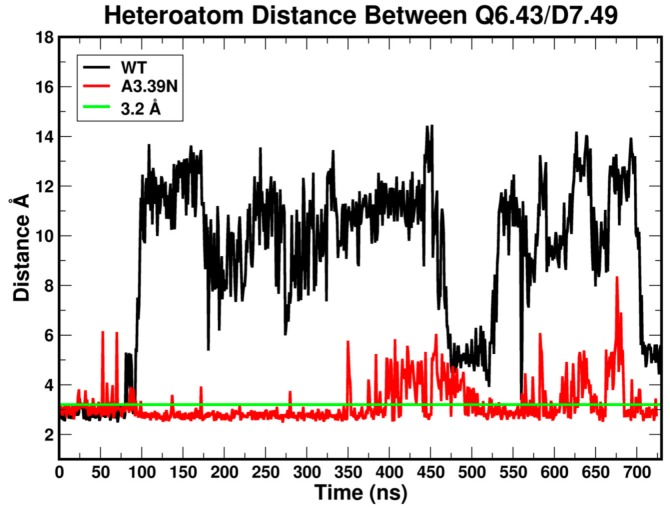
Heteroatom to heteroatom distance between residues Q6.43 and D7.49 in WT GPR18 (black) and the A3.39N mutant (red). The first 100 ns correspond to the equilibration phase. The next 600 ns are considered the production phase. The green line corresponds to 3.2 Å, the maximum distance to be considered a hydrogen bond.

The graphs in Figure 11 compare the heteroatom to heteroatom distances for the Q6.43-D7.49 pair. As shown in Figure 11, during the equilibration (first 100 ns), the distance is small enough for a hydrogen bond to exist between the two residues in the WT receptor (black line). This corresponds to the time frame in which D7.49 changes its conformation (Figure 9, black line). Past 100 ns, the WT distance is too great for a hydrogen bond to exist. In contrast, this distance is frequently below the maximum for a hydrogen bond in the A3.39N mutant.

**Figure 12:**
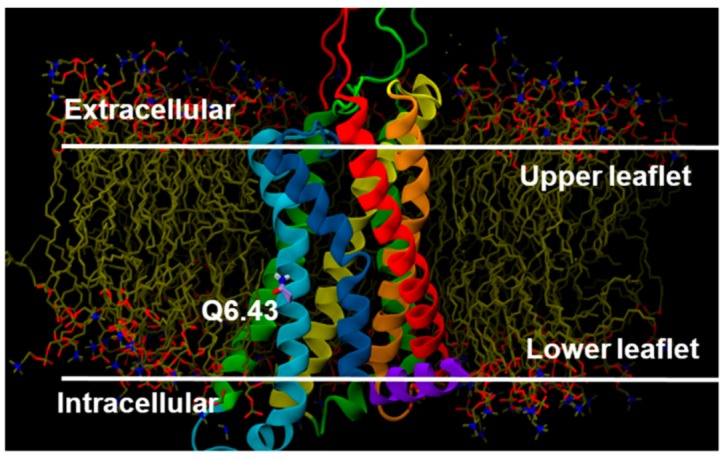
Position of Q6.43 in the GPR18 R model (highlighted in purple thin tubes).

Our MD simulations show that there is a strong relation between the Q6.43 to D7.49 hydrogen bond and the R3.50 to S6.33 “ionic lock” interaction. Even though glutamines are neutral, polar residues, based on our GPR18 model, Q6.43 is positioned so that it faces the lipid side of TMH6 (Figure 12). 

**Figure 13:**
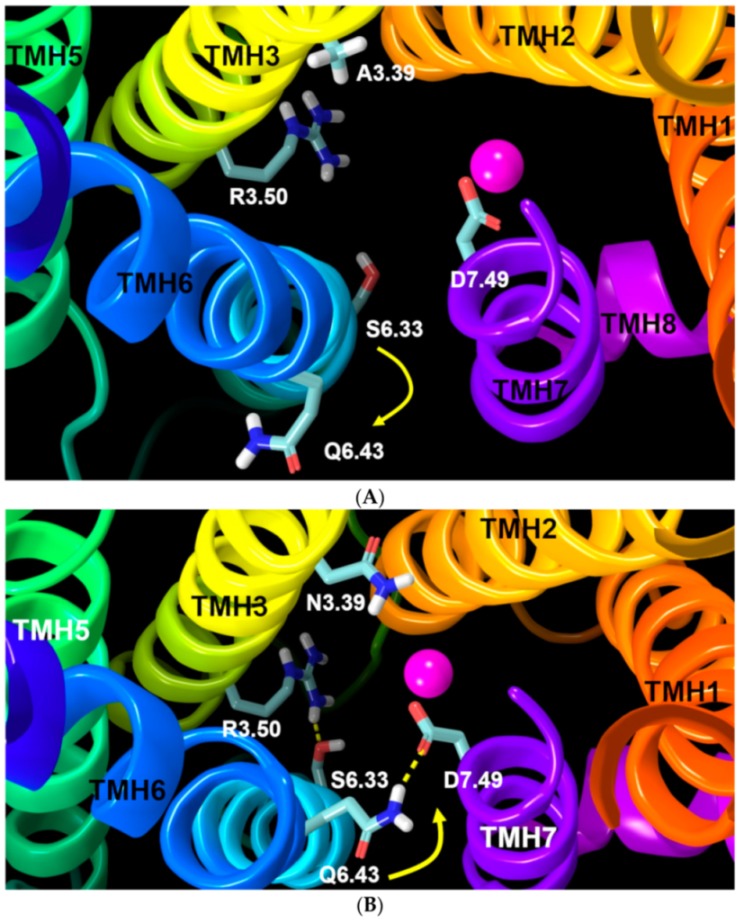
(**A**) Configuration of the important residues (highlighted in light blue tubes) involved in our proposed activation mechanism of GPR18. As D7.49 moves toward the sodium ion (magenta sphere) by shifting its χ1 from g^+^ to g^−^, the hydrogen bond between Q6.43 and D7.49 is broken. This leads to a clockwise movement (yellow arrow) of TMH6, which pulls it away from TMH3 and prevents the formation of the R3.50/S6.33 “ionic lock” in the WT receptor. (**B**) Configuration of the important residues (highlighted in light blue tubes) involved in the proposed activation mechanism in the A3.39N mutant. Hydrogen bonding occurs between Q6.43 and D7.49 (yellow dashed line) due to the sodium ion position (magenta sphere). This is facilitated by a counter-clockwise movement (yellow arrow) of TMH6. This movement facilitates the formation of the “ionic lock” hydrogen bond between R3.50/S6.33 (yellow dashed line) in the A3.39N mutant receptor.

Comparison of the Q6.43 sequence position with the corresponding residue in other class A GPCRs shows that the amino acids at this position are either non-polar residues (such as Val in CB1 and CB2, Ala in rhodopsin, Leu in adenosine A2A receptor) or small polar residues (like Thr in the β2-adrenergic receptor, or Ser in the formyl peptide receptor 2). Thus, having a long polar residue, like the glutamine, along with D7.49 is unique in the GPR18 sequence. MD trajectories reveal that Q6.43 plays an important role in the activation of the receptor in the absence of an agonist (i.e., constitutive activation). The driving force is the presence of the amide hydrophilic polar side chain of Q6.43 inside the hydrophobic non-polar acyl chain region of the phospholipid bilayer. As it can be seen in the trajectories, right after starting the simulation, Q6.43 tends to interact with nearby polar residues. Hydrogen bonding with D7.49 is the best and shortest way to do this. However, in the wild type system, this interaction is broken soon after the conformational change of the D7.49 χ1 from g+ to g^−^ (Figure 13A, Movie S1). This change leads to an active state bundle, which can hardly be seen in empty GPCR simulations (Movie S2). Our results suggest that the rotameric change of the D7.49 χ1 from g+ to g^−^ in the WT receptor causes the hydrogen bond between D7.49 and Q6.43 to break, which ultimately leads to a clockwise movement of TMH6 (from a top view) which will break the weak ionic lock between R3.50/S6.33 (Figure 13A).

Conversely, in the A3.39N mutant receptor, formation of the Q6.43/D7.49 hydrogen bond causes a counter-clockwise rotation (from the top view) of TMH6. This rotation brings S6.33 close enough to R3.50 to form an “ionic lock” hydrogen bond (Figure 13B, Movie S3). These results are in agreement with the experimental data that shows very high constitutive activity for GPR18 and reduced constitutive activity for the A3.39N mutant [15].

#### 2.2.3. Other Structural Features That May Contribute to High Constitutive Activity of WT GPR18

**Figure 14:**
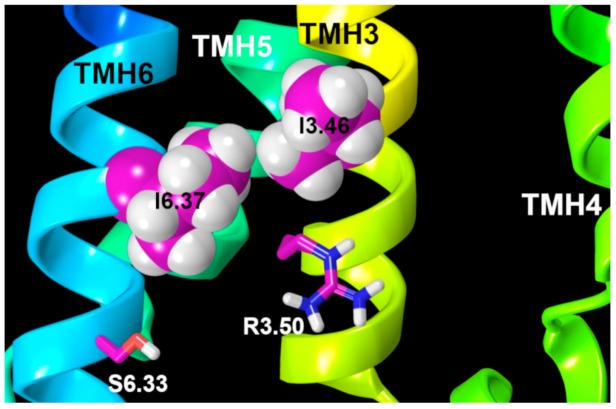
Presence of two bulky residues, I3.46 and I6.37 (magenta residues in van der Waals), above the ionic lock residues, R3.50/S6.33 (in magenta tubes), makes it more difficult to form a hydrogen bond in the WT receptor. This also likely contributes to the high basal activity of WT GPR18.

Further evidence of GPR18′s tendency to activate can be observed in the activated state model we developed. The first evidence is the quality of the ionic lock in GPR18, which is formed between R3.50 and S6.33. The ionic lock keeps TMH3 and TMH6 close at their IC ends and precludes G-protein interaction. In many other class A GPCRs, this interaction is formed between two charged amino acids, R3.50 and D/E6.30. Obviously, the interaction between a positively charged residue (arginine) and a negatively charged aspartic/glutamic acid is stronger than the interaction between the arginine and a polar neutral residue, like serine. Indeed, the “ionic lock” interaction in GPR18 is a hydrogen bond. Thus, it could be expected that this interaction in GPR18 could be easily broken. The second reason is the presence of bulky residues at the right top of the ionic lock. In the current GPR18 model, two isoleucines are located at the same level on TMH3 and TMH6 in the GPR18 bundle (see Figure 14). These residues may play an important role in the movement of TMH6. Several studies have suggested that residues at the relative positions of 3.46 and 6.37 play critical roles in GPCR activation [30]. Since these two residues are positioned above the ionic lock, the contacts between them might help to break the weak R3.50/S6.33 interaction in GPR18.

Similar structural features have been previously observed in other highly constitutively active receptors. The constitutive activity of GPCRs can be intensely affected by the sodium concentration, as well as by mutations in sodium-coordinating residues. For instance, in the chemokine receptors, mutation of key conserved residues related to the sodium pocket, including 2.50, 3.39, 7.45, and 7.49, has been shown to reduce the constitutive activity [17,18,19]. In this way, an A3.39S mutation in GPR18 may provide a broader mechanistic perspective of this orphan receptor. 

## 3. Materials and Methods 

### 3.1. Amino Acid Numbering System

The Ballesteros–Weinstein numbering system for GPCR residues is used in this paper. This system is based on assigning the label, 0.50, to the most highly conserved amino acid in class A GPCRs for each TMH [31]. This is preceded by the TMH number. In this system, for example, the most highly conserved residue in TMH4 is 4.50. The residue before this would be labeled 4.49, and the one placed immediately after this would be labeled 4.51. A residue in loops or the N- and C-termini is designated with its absolute sequence number. 

### 3.2. Receptor Model Development

A homology model of GPR18 was constructed using the X-ray crystal structure of the delta opioid receptor (DOR) at a 1.8 A° resolution as a template (PDB identifier: 4N6H). The DOR was chosen as the template because it has 26% sequence homology (similarity) with GPR18, has an analogous sodium binding pocket region, and has a key disulfide bridge between TMH3 and the EC-2 loop. Sequence similarities include key cysteines in the EC-2 loop (C(172) in GPR18; C(198) in DOR) that form a disulfide bridge with C3.25 on TMH3 (residues shown by double-headed arrow in Figure 1). This disulfide bridge helps limit the conformational flexibility of the EC-2 loop and stabilizes its structure. 

The GPR18 sequence was first aligned with the sequences of the Rho, CB1, CB2, DOR, β2-AR, and S1P1 receptors using the highly conserved class A GPCR residues/motifs as alignment guides (TMH 1 N1.50, TMH2 D2.50, TMH3 DRY motif from D3.49 to Y3.51, TMH4 W4.50, TMH5 P5.50, TMH 6 CWXP motif from C6.47 to P6.50 (blue residues in Figure 1), and TMH7 NPXXY motif from N7.49 to Y7.53). Based on this alignment, the DOR model was then mutated to the human GPR18 sequence. Two important motif variations in the human GPR18 sequence compared to other GPCRs in the alignment are (1) the presence of an F at position 6.48 in the TMH6 CWXP motif instead of W6.48, and (2) TMH7 motif variations with the GPR18 sequence being DVXXY vs. the common TMH7 motif, NPXXY. In addition, one critical residue difference in the GPR18 sequence is the presence of a proline in TMH3 at residue 3.36. The presence of this proline may bend TMH3. To explore these possible sequence-dictated conformational deviations, we employed the combined Monte Carlo (MC) and simulated annealing (SA) technique, conformational memories (CM). CM allows an exploration of the flexibility introduced by helix bending residues, such as proline, and outputs a set of low free energy helix conformations from which a new helix conformer can be chosen.

### 3.3. Conformational Memories Technique for Calculating TMH Conformations

Conformational memories is a simulated annealing/Monte Carlo (MC) conformational analysis method that can be used to explore the sequence dictated range of conformations available at biological temperatures (310 K) for a TMH. Key sequence divergences between the template and GPR18 were explored using CM. This technique has been previously validated in the development of GPCR models [32,33,34]. In this method, conformational consequences of helix bending residues were extensively and effectively explored in two separate phases. In the exploratory phase, the starting temperature for the simulated annealing (SA) was 3000 K, with cooling to 310 K in 18 temperature steps. At each temperature, 50,000 MC steps were applied. With each step, two dihedral angles and one bond angle were varied in the range of ±180° for dihedrals and of ±8° for bond angles. In the biased sampling phase, CM explores only regions that were highly populated in the first phase. The starting temperature was 749.4 K, with cooling in nine temperature steps to 310 K. At each temperature, 50,000 MC steps were applied. All calculations were performed using a distance-dependent dielectric and the PARAM22 force field [28,35,36] without CMAP corrections [37].

### 3.4. Modeling of the EC and IC Loop Conformations of the Receptor and the N- and C-Termini

The DOR crystal structure has its EC-2 loop splayed out, providing water molecules with free access to the binding crevice. Since the GPR18 endogenous ligands are lipid-derived, it is very likely that GPR18 ligands enter via the lipid bilayer [38]. As a result, the EC loops and/or the N-terminus likely shield the receptor binding pocket from water. Therefore, the GPR18 EC loops and N terminus were modeled so that they would shield the binding site from water. As Figure 1 shows, the EC-2 is the longest GPR18 loop (L(159)-V(184)) and it includes C(172), which likely forms a disulfide bridge with C3.25, providing some structural stability. MODELLER 8.2 [39,40,41] was used to generate the N terminus (Met(1) to Pro(18)) and the IC and EC loops (IC-1: Thr(51) to Thr(55), IC-2: Pro(126) to Lys(133), IC-3: His(216) to Val(226), EC-1: Glu(86) to Gly(90), EC-2: Leu(159) to Val(184), EC-3: Thr(259) to Tyr(264)). A limited distance between Cys(172) and Cys3.25 was placed in the EC-2 loop calculation so that the highly conserved disulfide bridge was preserved. The final loops were energy minimized to a gradient of 0.05 kJ/mol.Å with 2500 maximum iterations using the generalized born/surface area (GB/SA) continuum solvation model for water and the OPLS3 [42,43,44] force field in Macromodel 10.7.014 (Schrödinger Release 2017-4: MacroModel, Schrödinger, LLC, New York, NY, USA, 2017), while the TMH region was frozen.

### 3.5. Receptor Minimization

The resultant model was energy minimized using the protocol previously reported in our lab [45]. The minimization protocol used the OPLS3 [42,43,44] all atom force field in Macromodel (Schrödinger 2017-4: MacroModel, Schrödinger, LLC, New York, NY, USA, 2017). An extended Van der Waals cutoff (8.0 Å, updated every 10 steps), a 20.0 Å electrostatic cutoff, and a 4.0 Å hydrogen bond cutoff were used in each stage of the calculation. 

### 3.6. Molecular Dynamics (MD)

The wild type and mutant GPR18 models were oriented with respect to the membrane using the template, 4n6h, from the Orientation of Proteins in Membranes database [46] and placed in a hydrated 1-palmitoyl-2-oleoyl-sn-glycero-3-phosphocholine (POPC) bilayer using the CHARMM-GUI server. [47,48] The resulting system contained 155 POPC molecules, 15,177 water molecules, and an ionic strength of 0.15 M NaCl. The resulting simulation cell contained 71,363 atoms and was 80.3 Å × 80.3 Å 119.5 Å. All molecular dynamics runs employed the all atom additive CHARMM36m force field for proteins [49], and CHARMM36 for lipid and ions [28]. The system was energy minimized and relaxed using the multistep schedule as described previously [48]. Subsequent to this relaxation, 700 ns of unbiased MD was performed for each system. The first 100 ns of which were considered as equilibration (see Figure 3).

All MD simulations were performed with the GPU version of AMBER 16 [36,50,51] in the semi-isotropic NPT ensemble using a Langevin thermostat (*T* = 310 K, using a friction coefficient of 1 ps^−1^) and a Monte Carlo barostat (*P* = 1 bar) as implemented in AMBER 16. Long range electrostatics were treated using PME (particle mesh ewald) and the Van der Waal force switching method was applied starting at 10 Å with a cutoff of 12 Å. High-frequency bonds to hydrogen were constrained using the SHAKE method, allowing the use of a 2-fs integration time step.

Finally, a sodium ion was positioned in the putative sodium ion binding site. This site includes the polar residues, N1.50, D2.50, S3.39, N7.49, and Y7.53, and directly coordinates a sodium ion to stabilize the inactive state of the receptor. 

RMSD plots for the TMH bundle versus time were used to analyze the bundle geometry stability in the bilayer.

## 4. Conclusions

Our results suggest that the high constitutive activity of GPR18 is sequence driven and dependent on sodium binding pocket effects. To date, there has not been a systematic, comprehensive, and extended mutagenesis study of GPR18. Hopefully, by identifying the critical residues involved in the activation of GPR18, our model can aid future drug design studies. Further, we suggest that ligand development for GPR18 should concentrate on the development of inverse agonists/antagonists. It is possible that the sequence driven, high constitutive activity of GPR18 may preclude the need for agonist development.

## Supplementary Materials

Supplementary materials (Movies S1, S2, and S3 in Avi format) can be found at https://www.mdpi.com/1422-0067/20/9/2300/s1.

## Abbreviations

GPCR	G-Protein Coupled Receptor
NAGly	N-arachidonoyl glycine
POPC	Palmitoyloleoylphosphatidylcholine
CM	Conformational Memories
SA	Simulated Annealing
TMH	Transmembrane helix
DOR	delta opioid receptor
MD	Molecular dynamics
RMSD	root-mean-square deviation
EC	Extracellular
IC	Intracellular
WT	Wild type
AMBER	Assisted Model Building with Energy Refinement
